# Personality Trait Changes After Device-Aided Therapy: Insights From Parkinson's Patients and Their Close Relatives

**DOI:** 10.1155/padi/6681692

**Published:** 2025-07-25

**Authors:** Monica Scharfenort, Veronica Hernström, Kristina Rosqvist, Hanna Ljung, Maria H. Nilsson, Per Odin

**Affiliations:** ^1^Department of Clinical Sciences Lund, Faculty of Medicine, Lund University, Lund, Sweden; ^2^Department of Neurology, Rehabilitation Medicine, Memory and Geriatrics, Skåne University Hospital, Lund, Sweden; ^3^Department of Health Sciences, Faculty of Medicine, Lund University, Lund, Sweden; ^4^Memory Clinic, Skåne University Hospital, Malmö, Sweden

**Keywords:** carbidopa–levodopa drug combination, deep brain stimulation, infusion pumps, Parkinson's disease, personality, personality assessment, the Big Five Inventory

## Abstract

**Background:** It is unclear whether the two device-aided therapies (DATs: deep brain stimulation [DBS] and levodopa–carbidopa intestinal gel [LCIG]) affect personality in patients with Parkinson's disease (PD).

**Objective:** This retrospective study aims to explore if patients with PD and their close relatives experience any changes in the patient's personality following the start of DAT.

**Methods:** In total, 32 patients with DAT (17 DBS and 15 levodopa pump–based therapy) and their close relatives (*n* = 32) were asked to retrospectively assess potential personality changes in the patients using the Big Five Inventory (BFI) following DAT. They also answered questions regarding perceived quality of life and perceived impact on motor symptoms.

**Results:** There was a diverse perceived change in the patients' five personality traits when divided into the type of therapy. DBS patients reported decreased extraversion (70.6%, *n* = 12), while their close relatives reported an increase in the trait neuroticism (82.4%, *n* = 14). A significant difference was found in perceived changes in agreeableness, with 8 DBS patients reporting an increase and 4 close relatives reporting a decrease (*p*=0.008). Most of the pump patients (LCIG) reported a decreased conscientiousness (66.7% *n* = 10), a perception confirmed by 73.3% (*n* = 11) of their close relatives, who in addition reported an increase in neuroticism (73.3%, *n* = 11).

**Conclusions:** This study suggests perceived personality changes following DAT (DBS or LCIG), which vary by therapy and respondent role. Larger studies are needed, but the findings signal the importance of considering both patients' and their relatives' perspectives when assessing personality changes.

**Summary**

• Background
◦ It is not clear how the two advanced treatments for PD, DBS and LCIG, affect patient's personality.
• Objective
◦ This study explores if patients with PD or their close relatives notice any changes in the patient's personality after starting with the two treatments.
• Methods
◦ A total of 32 patients (17 with DBS and 15 with LCIG) answered a questionnaire called the BFI, where they looked back and reported any personality changes.
◦ They also answered questions about quality of life and the impact on motor symptoms.
• Results
◦ We found mixed results depending on the treatment type.
◦ For DBS patients, 12 patients (70.6%) reported being less outgoing (decreased extraversion).
◦ 14 relatives (82.4%) noticed more emotional instability (increased neuroticism).
◦ There was a notable difference in perceived agreeableness between patients and their relatives.
◦ For the LCIG patients, 10 patients (66,7%) reported being less impulsive (decreased conscientiousness) and 11 relatives confirmed this but also noticed more emotional instability (increased neuroticism).
• Conclusions
◦ This study suggests that the two treatments might lead to changes in patient's personalities that may vary depending on the treatment and who is reporting the changes.
◦ Larger studies are needed, but it is important to consider both patients and their relatives views when evaluating personality changes.

## 1. Introduction

Patients with Parkinson's disease (PD) are characterized by a multifaceted symptomatology, encompassing both motor (e.g., rigidity, tremor, and bradykinesia) and nonmotor impairments (e.g., urinary tract problems or mood disturbances). Clinicians have emphasized that no two patients with PD are the same [1]. Moreover, the complexity of the symptomatology makes treatment challenging in advanced stages of PD, which becomes evident when trying to determine the suitable device-aided therapy (DAT) for individual patients [2]. All forms of DAT, such as continuous infusion of apomorphine (CSAI), levodopa–carbidopa intestinal gel, with or without entacapone (LCIG/LECIG), foslevodopa/foscarbidopa, and deep brain stimulation (DBS), can significantly decrease motor complications [2] and nonmotor symptoms, including neuropsychiatric symptoms [3]. The association between reduced symptom severity and improved quality of life has been previously emphasized [4, 5]; however, there exist other variables impacting the quality of life, with personality being noteworthy [6]. To the best of our knowledge, it remains unclear how the DATs affect personality, which in turn can influence coping mechanisms and individual's perception of well-being [7].

Personality is defined as the enduring combination of characteristics that influence behavioral patterns and shape an individual's unique adaptation to life, including traits, values, self-concept, and emotional patterns [8]. One way to organize personality is according to the individual traits where the theory of the “Big Five” is normative with its five domains: extraversion (socially outgoing, able to experience positive emotions), agreeableness (yields to and trust others), conscientiousness (productive and organized), neuroticism (high anxiety, emotionally instable), and openness (willingness to explore in novel ideas, feelings, and experiences) [9]. Over the past decade, links between brain functions, structures, networks, and personality have been reported. However, in recent years, this association has been questioned, as existing research has proven difficult to replicate and has often yielded weak results [10]. Nevertheless, some studies suggest that treatments or interventions affecting the brain may lead to changes in personality. For instance, in children with epilepsy, normalization of personality traits has been observed following surgery [11]. Changes in personality after surgery for refractory epilepsy in adults have also been demonstrated [12]. In patients with cancer, one study reported personality changes after surgical resection, while another study found no such changes [13]. These mixed findings highlight the complexity of the relationship between brain interventions and personality outcomes. The impact on personality, following DAT, has focused on DBS therapy, wherein mixed results to personality changes have been reported [14]. Whether potential changes are permanent or transient is contested [15]. Regarding pump-aided therapies, the literature on personality traits is scarce despite their growing use in clinical practice. Understanding such changes is crucial, as personality traits are known to influence key aspects of life, including relationships, mental well-being, and quality of life. Moreover, given the potential for limited introspective ability in patients, involving close relatives can provide valuable insights [16]. Consequently, this study aimed to retrospectively explore perceived personality changes following DBS and levodopa-based pump therapy, capturing perspectives from both patients and their close relatives. By addressing this gap, the study contributes to a deeper understanding of personality changes following DAT, offering insights to both theory and clinical practice. This knowledge can help clinicians tailor support for patients and their relatives, who may find such changes challenging. By guiding patients toward realistic expectations regarding personality changes throughout the course of treatment, clinicians may help prevent negative and potentially distressing reactions to unmet expectations.

## 2. Materials and Methods

### 2.1. Study Design, Participant Recruitment, and Consent

A prior retrospective study from 2016/2017 [17] included 67 advanced patients with PD who received DBS, CSAI, or LCIG. Participants were recruited from three sites: Skåne University Hospital and Uppsala University Hospital in Sweden and Bispebjerg University Hospital in Copenhagen, Denmark. The participants were identified either through their consulting neurologists or through the Swedish Parkinson patient registry, ParkReg (part of Swedish Neuro Registries, see https://www.neuroreg.org), with a national coverage rate of 70% for patients with advanced therapies. ParkReg facilitates the identification of suitable patients for clinical studies based on the study's inclusion/exclusion criteria. The inclusion criteria in this study were patients with idiopathic PD (G 20.9) with at least a year of DAT (DBS, LCIG, and CSAI) by the study's commencement and being 67 or younger when the therapy began. Participants were prompted to include a relative, preferably a spouse or other close relative. Exclusion criteria were severe dysarthria or dementia in the patient or the partner.

In the present retrospective study, the same 67 patients and their selected relatives were invited in 2017/2018 to complete a modified, retrospective Big Five Inventory (BFI) questionnaire [18, 19]. It was sent by mail and followed by a two-week reminder call, and 39 questionnaires were returned. After excluding twelve due to insufficient data, and the three patients treated with apomorphine, 24 patient-relative pairs were incorporated into the present study. Furthermore, we incorporated 8 patients from a subsequent study titled “Advanced therapy in PD from a patient and close relative perspective,” where the inclusion criteria were nondemented patients awaiting the start of DAT. The participants were also in this study prompted to include a relative. Both patients and their close relatives completed the retrospective BFI during the period from 2023 to 2024.

The study protocol was reviewed by the Regional Ethical Review Board at Lund University, Sweden (nr: 2017/8). No ethics application was needed in Denmark due to the study's design. Written informed consent was obtained from all included participants.

### 2.2. Personality Assessments

The BFI, which is based on the five factor theory of personality [20], was used to evaluate changes in personality. It includes 44 items that assess five personality traits: openness (10 items), conscientiousness (9 items), extraversion (8 items), agreeableness (9 items), and neuroticism (8 items). The BFI was modified for a retrospective approach to assess perceived personality changes after the start of DAT. The modification consisted of a change in the response options where both the patients and their close relatives assessed whether the 44 characteristics of the BFI had increased, decreased, or remained unchanged in the patient following DAT.

### 2.3. Additional Descriptive Data Collection

Patients and their close relatives were also asked if they perceived that, following DAT, the patient's quality of life and motor symptoms had changed. Both questions were answered using a 5-point Likert scale: 1 = strongly worsened, 3 = the same, and 5 = strongly improved (i.e., 2 and 4 were undefined).

### 2.4. Data/Statistical Analyses

The responses to BFI were given the following values: −1, if the characteristic behavior was considered to have decreased (“worsened”); 0, if it was perceived as unchanged; and +1, if it was perceived as increased (“improved”). Dimension scores were created by averaging all values for each dimension, ensuring that all participants got scores between −1 and +1. Data on personality traits are presented as increased trait (total sum is positive), the same (total sum is null), or decreased trait (total sum is negative). The results divided by the therapy group and participant role are shown in total numbers and percentages.

Statistical differences were evaluated using the Wilcoxon signed-rank test (perceived personality changes). *p* value < 0.05 was considered statistically significant. All analyses were conducted using IBM SPSS Statistics for Windows (Version 28). One participant (patient) had missing data in two out of the 44 questions. Accordingly, as per the recommendations of the BFI author, the average trait domain was calculated (without the missing data) for the participants group; second, the missing data were replaced with this average answer [21].

## 3. Results

A total of 32 patients treated with DBS or LCIG as well as their close relatives responded to the BFI questionnaire retrospectively. Seventeen patients had DBS (10 men and 7 women), and 15 had LCIG (10 men and 5 women) (see Table 1).

The median age (q1–q3) of the DBS patients was 64 (57.5–66.5) years, and that of the pump patients was 69 (64.0—69.0). The median (q1–q3) years with DAT (i.e., time between start of DAT and inclusion to this study) was 4 years (3.0–6.5) for DBS patients and 0.5 years (0.5–6.0) for pump-aided therapy (see Table 1). The close relatives consisted of 28 spouses (17 wives and 11 husbands) and 4 children (see Table 1).

### 3.1. Personality Changes Following DAT Divided by Therapy

**Figure 1:**
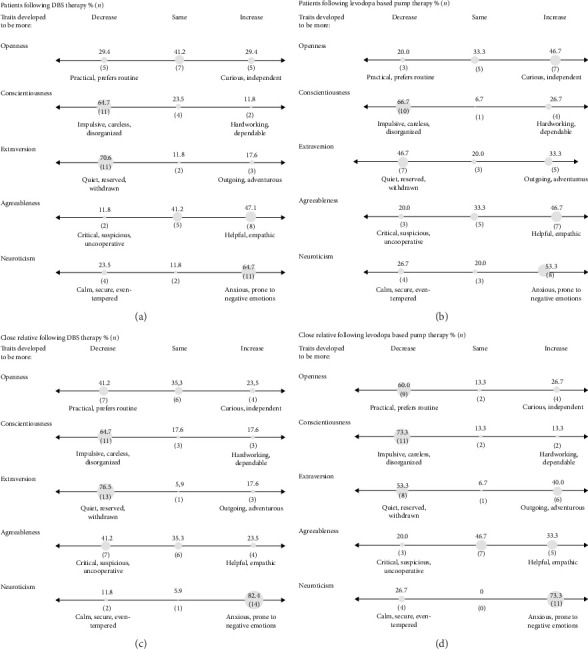
Changes in patient's Big Five Inventory following device-aided therapy: Perspective of patients and close relatives; values are reported as numbers and percentages. 32 participants are included in total, 17 DBS and 15 levodopa pump–aided therapy. Changes in Big Five Inventory (BFI) scores: increase, same, decrease, following device-aided therapy (DBS: deep brain stimulation, or levodopa pump–aided therapy and levodopa–carbidopa intestinal gel with or without entacapone). BFI includes the 5 dimensions: openness (willingness to explore novel ideas, feelings, and experiences), conscientiousness (productive and organized), extraversion (socially outgoing, able to experience positive emotions), agreeableness (yields to and trusts others), and neuroticism (high anxiety, emotionally unstable). (a and b) The patients' perceived changes in their own BFI. (c and d) The close relatives' perception of the patients' changes in BFI. Percentages may not total 100% due to rounding.

The personality changes in the patients following DAT show more similarities than diversity comparing who is reporting the perceived personality change, as shown in Figures 1(a) and 1(b). The DBS patients perceived the biggest shifts in the personality trait extraversion (70.6%, *n* = 12 reported a decrease), followed by a shift in both neuroticism (increased by 64.7%, *n* = 11) and conscientiousness (decreased by 64.7%, *n* = 11). The close relatives of DBS instead predominantly reported an increase in neuroticism trait (82.4% *n* = 14), followed by a decrease in extraversion (76,5%, *n* = 13).

Among the LCIG patients, the biggest shift was reported in both the trait conscientiousness, with 66.7% (*n* = 10) reporting a decrease, followed by an increase in neuroticism (53.3% *n* = 8). Relatives reported similar changes, with equal rates (73.3%, *n* = 11) of decreased conscientiousness and increased neuroticism.

Comparing the close relatives' versus the patients' perceptions shows similarities and disparities (see Figure 1). The difference was statistically significant only in the trait agreeableness, and only among DBS couples (*p*=0.008); eight patients (47.1%) reported a perceived increase, compared to a perceived decrease reported by seven of the close relatives (41.2%).

### 3.2. Changes in Perceived Motor Symptoms and Quality of Life Following DAT

Following DAT, 66.7% (*n* = 10) of the pump patients and 47.1% (*n* = 8) of those with DBS reported an improvement of their motor symptoms (see Table 2). There was a reported improvement of quality of life following DAT by 80% (*n* = 12) of the pump patients and 41.2% (*n* = 7) of DBS patients (see Table 2).

## 4. Discussion

This study aimed to explore retrospective perceptions of personality changes following DAT in 32 patients with PD, from both the patient's and close relatives' perspectives. The results reveal that both the patients' own perceptions of change and those of their close relatives varied depending on the type of therapy.

Regarding the DBS couples, the main finding is their shared perception of major changes in the three traits of extraversion, conscientiousness, and neuroticism. Both patients and close relatives reported that following DBS, the patients became more withdrawn, reserved, and less extraverted. A decrease in self-discipline (decreased conscientiousness) and heightened emotional responsiveness (increased neuroticism) were also reported. The subjective decrease in conscientiousness following DBS may be partly explained by changes in executive function after DBS, i.e., as this personality trait has been closely linked to executive functions [22, 23].

The current study also showed discrepancies between patients and relatives in relation to some traits. The largest proportion of DBS patients reported a decreased extraversion, followed by an increased neuroticism. In contrast, relatives most frequently reported increased neuroticism, followed by decreased extraversion. Decreased extraversion and high neuroticism have been described as a specific PD personality profile [24]. There is a hypothesis that DBS might stimulate specific brain regions associated with distinct personality traits; the medial temporal lobe and basal ganglia are linked to neuroticism, whereas the medial orbitofrontal cortex is linked to extraversion [25, 26]. Still, this profile has also been described in various other psychopathological disorders such as depression and alcohol abuse [27]. It should be noted that a decreased conscientiousness in combination with increased neuroticism may negatively affect coping, and it may induce a sensitivity to psychological distress [28]. All considered, these results underscore the need for longitudinal observations to determine whether the changes are associated with PD, the concomitant disorders, the individual's personality, age-related changes in personality or DAT. It might also be of interest to also mention the results from Boussac et al. [29], who stated that PD patients awaiting DBS operation had a significant, distinctly different characteristic personality trait compared to age-matched normative subjects. The authors suggested that awaiting a possible stressful surgery may be a cause for the personality differences observed in the patients awaiting DBS [29].

Finally, the DBS couples reported a change regarding agreeableness. Patients reported a higher level of helpfulness and empathic feelings, whereas close relatives instead reported a decreased level, including more suspicion. This created a significant difference within the DBS couples. It has been suggested that those with higher agreeableness are more likely to listen to their relatives and recognize the benefits of the treatment. Higher agreeableness has also been associated with better quality of life and greater adaptability to coping strategies [28]. Conversely, lower agreeableness is linked to mental health issues, including treatment-resistance depression [30, 31].

The main findings regarding couples with LCIG were the extent of change rather than the trait itself, which varied between patients and their close relatives. The patients reported that they were more impulsive, disorganized, and had a decreased conscientiousness. Their close relatives confirmed these changes, including an increased anxiety and being more prone to negative emotions (increased neuroticism). These findings align with previous studies of LCIG, which noted emotional instability (increased neuroticism) [16, 32]. Interestingly, in contrast to DBS patients, LCIG patients tend to see themselves as becoming more curious (increased openness) and adventurous (increased extraversion). Individuals with higher levels of openness are often more creative, flexible, and use humor to cope with stress. When combined with agreeableness and extraversion, openness is linked to better coping skills and improved mental health [28].

In summary, all the participants agreed that following DAT, patients exhibited decreased conscientiousness and increased neuroticism. Research indicates that all personality traits can influence lifestyle factors, such as stress sensitivity, substance abuse, depression, and sleep patterns, which may, in turn, affect physical, mental, and social well-being. This aligns with previous research linking personality traits to various health conditions [33–35] and increased mortality risk [36]. Furthermore, several neurological disorders have been associated with different personality traits, such as elevated neuroticism [24, 37]. This highlights a dual connection: it remains unclear whether these personality traits emerge because of the neurological disease's impact on brain structure, chemistry, and function, or whether individuals already possess these traits prior to developing the condition. For example, Kang et al. [38] demonstrated that high conscientiousness and openness were associated with a reduced risk of developing a multiple sclerosis diagnosis 7 years later. In people with PD, high neuroticism is negatively associated with quality of life, while high conscientiousness is positively associated [6]. In the present study population, characterized by increased neuroticism and decreased conscientiousness, these changes could contribute to reduced quality of life, observed in 71% of DBS patients and 50% of LCIG patients.

As personality traits are associated with coping styles [39], they may influence the perceived outcome of treatment, despite the clinical improvement. In the present study, 66.7% (*n* = 10) of LCIG patients reported improvements in motor symptoms following DAT, compared to 47.1% (*n* = 8) of DBS patients. This difference may be influenced by factors such as time since DAT initiation (ranging from 0.5 to 6.5 years), dose adjustments of peroral dopamine treatment, as well as patients' levels of agreeableness, potentially affecting their recall of treatment benefits. Personality traits have been associated with variations in executive functioning and memory processes, both of which are known to influence the reconstruction of autobiographical memories and the subjective evaluation of treatment outcomes [40, 41]. Although studies specifically linking personality traits and motor symptomatology in PD are limited, available evidence indicates that motor fluctuations may correspond to concurrent changes in personality dimensions [29]. An association between decreased mobility and higher neuroticism levels has been reported in PD patients [42]. Interestingly, DBS patients have shown improved motor function alongside increased neuroticism [32]. Research on personality changes in pump-aided therapy is limited but, in the present study, increases in both neuroticism and improved motor function were observed in the LCIG patients. It is unclear whether these changes are due to the disease process or the dopaminergic therapy. Notably, treatments for neurological conditions have also been shown to influence personality. For instance, epilepsy patients who became seizure-free exhibited lower neuroticism and higher openness, conscientiousness, and extraversion scores compared to those with seizures, a change attributed to the recovery of psychophysiological pathways [37]. Dopaminergic supplementation, known to promote exploratory behavior and novelty seeking, both linked to extraversion, may also affect personality. Since dopaminergic medication is typically reduced after DBS, this change may partly explain the reported reduction in extraversion [21]. In contrast, continuous dopaminergic delivery with LCIG allows for stable plasma levels and monotherapy, potentially reducing nonmotor symptoms such as mood and sleep disturbances [43–45]. That said, the personality changes may be influenced by medication adjustments and improvements in nonmotor symptoms.

Given this complex interplay between personality traits and neurological conditions, our findings on personality changes following DAT contribute important insights. Patients undergoing DAT may experience ongoing personality changes, due to disease progression per se as well as psychological reactions. These changes, although not fitting standard psychiatric definitions, are significant concerns for patients and their relatives. In addition, personality traits may influence the choice and perceived expectations of DAT [29]. It may therefore be important to monitor personality in patients awaiting and following DAT to evaluate its outcomes effectively.

Finally, as seen in the present study, a difference is often noticed in how patients and their caregivers view personality changes. For instance, patients may feel “happier,” while relatives may view them as “too impulsive.” This highlights the importance of involving close relatives in the assessment process. Further studies comparing personality traits before and following DAT from both patient's and close relatives' perspectives are needed.

### 4.1. Strengths, Limitations, and Future Perspectives

To the best of our knowledge, this is the first study to address personality change following both DBS and pump therapy. The inclusion of close relatives possessed important strengths, confirmed by the observed differences in perceived personality changes between patients and caregivers that are presented in the study. Another strength is that we performed a standardized evaluation of personality using the BFI.

A limitation of our study is the sample size. Another limitation is the retrospective design, particularly because the mean timeframe between initiating DAT and the data collection ranged from 0.5 to 6.5 years. A prospective study may yield different results following DAT with more accurately reflected personality prior to and following DAT. The lack of randomization among the patients makes it inappropriate to compare the results between the different therapies. It should also be noted that individual factors related to the treatment procedure, such as electrode trajectory and placement in DBS, the design of stimulation parameters, or how pump therapy is dosed, may potentially influence personality outcomes. However, investigating these aspects would require larger and more rigorously controlled studies. Another limitation of the study design concerns the modified scale used in the BFI. The Swedish version of the BFI has previously demonstrated good reliability (Cronbach's alpha = 0.73–0.84) and a clear factor structure [46]. However, in this study, the response scale was modified to fit the retrospective design, meaning that the reliability and validity of the current version would need to be further assessed separately.

Other limitations include that we did not register the number of participants who were excluded due to the exclusion criteria. Moreover, 30 participants out of the original 67 declined to participate. In addition, 12 participants were excluded due to having insufficient data. These aspects may affect the external validity of our findings, which should be kept in mind when interpreting the results.

Future studies should also include documentation of concomitant medication since these treatments can affect the personality. Moreover, more detailed descriptive information (e.g., motor symptoms) would be of value. Lastly, there are unfortunately numerous personality models, each utilizing its own set of instruments. This makes it challenging to compare the reported results across different models. It is crucial to recognize that despite potential similarities, each model is constructed within its own unique theoretical framework. In addition, these models offer distinct perspectives on personality, emphasizing the importance of considering their respective findings.

## 5. Conclusions

This study suggests that both patients and relatives experience personality changes following DAT, although the experiences partly differ between patients and relatives. In summary, all participants agreed that there was a decrease in conscientiousness and an increase in neuroticism, although the extent varied between different therapies and populations. This suggests that patients, overall, exhibit more impulsiveness and a tendency to interpret situations more negatively, potentially impacting both the quality of life and the experience of DAT. Personality changes can probably have a strong impact on both the patient and his/her spouse/close relatives, which is why further larger prospective studies on these effects are highly warranted.

## Figures and Tables

**Table 1:** Demographic characteristics of study cohort (*n* = 32)^a^.

	Deep brain simulation (DBS)^b^*n* = 17	Levodopa-based pump therapy^c^*n* = 15
Sex, female/male (*n*)	7/10	5/10
Age, y (median, q1–q3)	64.0 (57.5–66.5)	69 (64.0–69.0)
DAT ^d^ duration, y since start of DAT (median, q1–q3)	4 (3.0–6.5)	0.5 (0.5–6.0)
Close relative (*n*)		
Wife	9	8
Husband	6	5
Child	2	2

^a^Values are reported as median and first and third quartiles (q1–q3) and numbers and percentages. 32 participants are included in total.

^b^DBS: Deep brain stimulation.

^c^Levodopa-based pump therapy: Levodopa–carbidopa intestinal gel with or without entacapone.

^d^DAT, Device-aided therapy, i.e., DBS or pump.

**Table 2:** Clinical characteristics of the study cohort^a^.

	Deep brain simulation (DBS)^b^*n* = 17		Levodopa-based pump therapy^c^*n* = 15	
	Patient	Close relative	Patient	Close relative
Changes in motor symptoms following DAT ^d,e^ (*n*, %)				
Improvement	8 (47.1)	7 (41.2)	10 (66.7)	10 (66.7)
No change	2 (11.8)	4 (23.5)	3 (20.0)	3 (20.0)
Worsening	7 (41.2)	6 (35.3)	2 (13.3)	2 (13.3)
Changes in quality of life following DAT^f^ (*n*, %)				
Improvement	7 (41.2)	5 (29.4)	12 (80)	11 (73.3)
No change	4 (23.5)	5 (29.4)	2 (13.3)	1 (6.7)
Worsening	6 (35.3)	7 (41.2)	1 (6.7)	3 (20.0)

^a^Values are reported as median and first and third quartiles (q1–q3) and numbers and percentages. 32 participants are included in total.

^b^DBS: Deep brain stimulation.

^c^Levodopa-based pump therapy: Levodopa–carbidopa intestinal gel with or without entacapone.

^d^DAT, Device-aided therapy, i.e., DBS or pump.

^e^Changes in motor symptoms, the patient's or close relative's perception of change in the patient's motor functioning following DAT (worse, same, and improvement).

^f^Changes in quality of life, the patient's or close relative's perception of change in the patient's quality of life following DAT (worse, same, and improvement).

## Data Availability

The data that support the findings of this study are available on request from the corresponding author. The data are not publicly available due to privacy or ethical restrictions.
